# Relationship Between Stressful Life Events and Depression Among Adolescents: The Mediating Roles of Subcomponents of Executive Function

**DOI:** 10.3390/bs15020145

**Published:** 2025-01-29

**Authors:** Wenli He, Qiong Hu, Jiejie Wang, Yingbo Rao, Chen Cheng, Ping Fang, Qiong Zhang, Yunrong Lu

**Affiliations:** 1 Department of Psychiatry, The Fourth Affiliated Hospital of School of Medicine, and International School of Medicine, International Institutes of Medicine, Zhejiang University, Yiwu 322000, China; 8021057@zju.edu.cn (W.H.); wangjiejie0914@163.com (J.W.); 8615252@zju.edu.cn (P.F.); 2 Department of Psychology and Behavioral Sciences, Zhejiang University, Hangzhou 310000, China; huqiongpsy@zju.edu.cn (Q.H.); zhangqiongzgh@zju.edu.cn (Q.Z.); 3 Department of Clinical Laboratory, The Fourth Affiliated Hospital of School of Medicine, and International School of Medicine, International Institutes of Medicine, Zhejiang University, Yiwu 322000, China; ryb1987@zju.edu.cn (Y.R.); 8014041@zju.edu.cn (C.C.)

**Keywords:** depression, adolescence, stressful life events, executive functions

## Abstract

Stressful life events are important risk factors in the development of adolescent depression. Executive function is significant in the stress–depression link. However, it is not clear whether there is a specific effect for subcomponents of executive function (working memory, inhibition, and shifting). Therefore, the present study recruited 213 adolescents (mean age (*M*_age_) = 15.19 years, SD = 1.27, range = 12.00–18.00 years, and 53.00% girls) and measured their perceived stress using the questionnaire of the Adolescent Self-Rating Life Events Checklist, working memory ability by two-back tasks, inhibition ability by Stroop tasks, and shifting ability by Wisconsin Card-Sorting tasks. Results showed that stressful life events positively correlated with adolescents’ depression, while stressful life events negatively linked with working memory and inhibition. Depression was negatively associated with working memory and inhibition. No significant correlation was found between shifting and either stressful life events or depression. Mediation analyses revealed that working memory and inhibition mediated the link between stressful life events and adolescent depression, while shifting did not show a mediating effect. Our findings provide further evidence for the precise effect of executive function in the stress–depression link, implicating that different subcomponents should be considered to provide targeted intervention to alleviate adolescents’ depressive symptoms.

## 1. Introduction

Depression is a major source of mental and physical problems and suffering in adolescence (Shorey et al., 2022). It is also considered to be an important remediable risk factor for suicide (Johnson et al., 2018). Depression can have significant effects on adolescents’ school performance, their interpersonal functions later in life, early parenthood, and it can increase the risk of other mental health disorders (Birmaher et al., 2007; Cheung et al., 2013). The prevalence of depression has increased across all age groups worldwide, and this increase among adolescents has outpaced that among adults (Weinberger et al., 2018). Thus, understanding the etiology of adolescent depression is of great importance for formulating prevention and treatment strategies.

### 1.1. Stressful Life Events and Depression

As the diathesis–stress model suggests, individuals are likely to develop a mental disorder if they are exposed to stressful life events (Cruz-Pereira et al., 2020; Hammen, 2005, 2018; Malhi & Mann, 2018; Monroe & Simons, 1991). Cognitive theories of depression further stress the role of cognitive processes and how their deficits influence individuals to develop affective symptoms of this disorder (Joormann & Quinn, 2014; LeMoult & Gotlib, 2019, for a review). However, as a domain of cognitive function, the mediating role of executive function in the relation between stressful life events and depression has not been fully investigated.

### 1.2. Stressful Life Events and Executive Function

Executive function refers to a set of cognitive processes that allow individuals to plan, regulate thoughts and actions, and eventually achieve their goals. It has been argued that inhibitory control, working memory, and cognitive flexibility are the most fundamental functions underlying higher cognitive functions (e.g., Diamond, 2013; Ishihara et al., 2021). A plethora of studies reported that executive function may be influenced by stress. For instance, executive function deficits were found in participants aged 9 to 18 years who experienced high levels of early life stress (van Rijn et al., 2018; Y. Zhang et al., 2019). Adolescents with complex trauma experiences and prolonged institutional care were reported to exhibit more deficits in executive function (Katembu et al., 2023; Op den Kelder et al., 2017). Evidence from longitudinal studies confirmed that, if these individuals suffered more cumulative negative life events during their growth period, they showed less efficient executive function in their early adulthood (Heyman & Hauser-Cram, 2015; Wade, 2023).

### 1.3. Executive Function and Depression

Research has also demonstrated that depression is associated with a deficiency in executive function. Particularly, executive function in early childhood was found to be predictive of depression across school age, with less efficient performance in executive function relating to more severe depressive symptoms (Hawkey et al., 2018). Targeted training on executive function has been shown to considerably reduce self-reported depression in adolescents, which maintained for one month (Beloe & Derakshan, 2020).

### 1.4. The Mediating Role of Executive Functions

Taken together, stressful life events can lead to a depletion of executive function, which may, in turn, increase the risk of depressive symptoms. This indicates that executive function may serve as a pathway linking stressful life events to depression, which has been supported by empirical evidence. For instance, stress-induced declines in executive control have been shown to predict depressive symptoms (Quinn & Joormann, 2015), and executive dysfunction has been reported to mediate the relationship between adverse childhood experiences and mental distress (Trossman et al., 2021). Moreover, executive dysfunction was reported to serve as a mediating factor that exacerbated depression under the stress associated with the COVID-19 pandemic (Hong et al., 2023).

However, depression is a highly heterogeneous syndrome with varying genetic, neurophysiological, and psychological mechanisms contributing to diverse symptoms, clinical trajectories, and treatment outcomes (e.g., Lynch et al., 2020). This heterogeneity underscores the importance of investigating the distinctive roles of specific subcomponents of executive function, especially working memory, inhibition, and shifting, in linking stress to depression. Specifically, impairments in inhibition and working memory were shown to link with depression (e.g., García-Martín et al., 2021; Nikolin et al., 2021), while deficits in shifting, rather than inhibition, were reported to correlate with depression (Conner et al., 2023; Sun et al., 2023). In addition, stressful life events have been found to differentially affect working memory (Shields et al., 2017), inhibition, and shifting (Gabrys et al., 2018; Shields et al., 2016). Given the inconsistency, we speculated that the mediating roles of subcomponents of executive function in the relation between stressful life events and depression may differ from each other.

### 1.5. Potential Confounder

To note, gender might confound the relation between stressful life events, executive function, and depression. Since the brain demonstrates different developmental trajectories between genders, this may lead to varying outcomes when encountering stressful life events (Pechtel & Pizzagalli, 2011). Specifically, males exhibit less vulnerability to depression compared to females (Nolen-Hoeksema, 2001; Snyder, 2013). Also, boys tend to excel in working memory tasks requiring mental counters (Wierenga et al., 2019).

Further, grades also affect the association between stressful life events, executive function, and depression. For instance, the incidence rates of depression have been found to vary across different grades, with the overall risk of depression incidence being higher in the group aged 15–19 years than those aged 10–14 years (Yang et al., 2024). Thus, in the present study, both gender and grade were included as covariates in the analysis.

### 1.6. The Present Study

To summarize, although it is accepted that executive function mediates the relationship between stressful life events and depression, how different components of executive function mediate the association between stressful life events and depression remains open. Thus, the primary objective of the present study was to fill this gap and thus provide a more comprehensive insight into the cognitive mechanisms underlying stress-related depression.

## 2. Methods

### 2.1. Participants and Procedure

A total of 213 adolescents (*M*_age_ = 15.19 years, *SD* = 1.27, range = 12.00–18.00 years, 53.00% female) were recruited from Hangzhou in Southeast China between 2022 and 2023. Among them, 115 adolescents (*M*_age_ = 14.96 years, *SD* = 1.33, range = 12.00–18.00 years, 68.70% female) were diagnosed with depressive disorder by psychiatrists from the psychiatric department at an affiliated hospital of one of the top five universities of China. The exclusion criteria included patients with severe somatic diseases and those with brain-organic mental disorders, schizophrenia, or bipolar disorder. Another group of 98 healthy adolescents (*M*_age_ = 15.46 years, *SD* = 1.13, range = 13.00–17.00 years, 34.69% female) were from a middle school.

All participants were instructed to independently complete two questionnaires and finish three executive function tasks using E-Prime 2.0 software in privacy in a separate room (Psychology Software Tools, Pittsburgh, PA, USA). This study was approved by the Human Research Ethics Committee of the authors’ institution. All participants and their parents signed a written informed consent before taking part in the study.

### 2.2. Materials

#### 2.2.1. Stressful Life Events

The Adolescent Self-Rating Life Events Checklist is widely used to assess the frequency and stress intensity of life events among Chinese adolescents (Liu et al., 1997). It consists of 27 items assessing six domains: interpersonal relationships, academic pressure, punishment, loss, healthy adaptation, and other factors. Higher scores indicate a greater impact of negative life events on adolescents. In the current study, the questionnaire demonstrated a high internal consistency reliability coefficient (Cronbach’s alpha = 0.95).

#### 2.2.2. Executive Function

Two-back task (adapted from Jaeggi et al., 2011). This task measures working memory. In this task, a cartoon monkey king was positioned randomly in a 3 × 3 grid on the screen. In each trial, participants were required to press the key as quickly as possible to judge whether the displayed stimulus was at the same position as the one presented two trials earlier. If the position was the same, the participant had to press the A key; otherwise, the L key. The two-back task was divided into two blocks, each with 20 trials. The trial order in the block was pseudorandomized so it was equal for all participants. The accuracy of the two-back task was used to measure the working-memory ability of the participant. This task requires participants to hold information in mind for short durations, continuously refresh the contents in the storage system with each new stimulus, and compare the current stimulus with one presented two steps earlier. These processes collectively reflect the dynamic nature of working memory and make this task a comprehensive tool for assessing working-memory capacity (Li et al., 2024).

Stroop task (Zhang et al., 2019). This task measures individuals’ inhibition. In this task, different numbers of cherries and watermelons were shown on the left and right sides of the screen in each trial. Participants were asked to neglect the fruit size and rapidly judge which side of the screen contained more fruits. The rule was to press the A key when the left screen presented more fruits than the right, or the L key when the right screen showed more. A total of 80 trials were set, including 40 congruent trials (the number of fruits with larger sizes was higher than that of smaller ones, e.g., four watermelons versus two cherries) and 40 incongruent trials (the number of fruits with larger sizes was lower than that of smaller ones, e.g., four cherries versus two watermelons). Longer reaction times of incongruent trials indicated poorer inhibitory control ability. The Stroop task is widely used to measure inhibitory control because it directly involves resolving interference between two competing responses (number of fruits and size of fruits), which is a core feature of inhibitory control. This mechanism closely parallels the cognitive challenges faced by individuals with depression when dealing with negative events or intrusive thoughts (Gligorović & Buha, 2014).

Wisconsin Card-Sorting Task (Heaton & Psychological Assessment Resources Staff, 2003). This task measures individuals’ ability to shift. In this task, participants were engaged in a task where they had to match response cards to stimulus cards depicting figures with variations in shape, color, and number. Importantly, they were not provided with explicit instructions regarding the sorting criteria. After each attempt, they received feedback indicating whether their response was correct or not. The sorting rule was modified once children successfully matched the cards for 10 consecutive trials. Participants might make errors once the rule changes, indicating difficulty in adapting to the new rule. A greater number of perseverative error trials indicates a diminished ability to shift. This task is an ideal tool for assessing shifting ability, as it demands participants integrate rules, inhibit automatic responses, and infer abstract patterns (Larsen & Luna, 2018). It is particularly suitable for adolescents, whose maturation of the prefrontal cortex enhances higher-order cognitive abilities (Larsen & Luna, 2018). Further, multiple dimensions, including color, shape, and number, require participants to engage simultaneously in rule acquisition, hypothesis testing, and error correction. This multifaceted structure provides a nuanced and comprehensive assessment of shifting and problem-solving abilities, making the WCST a highly reliable and ecologically valid measure for evaluating cognitive flexibility in adolescents.

**Figure 1:**
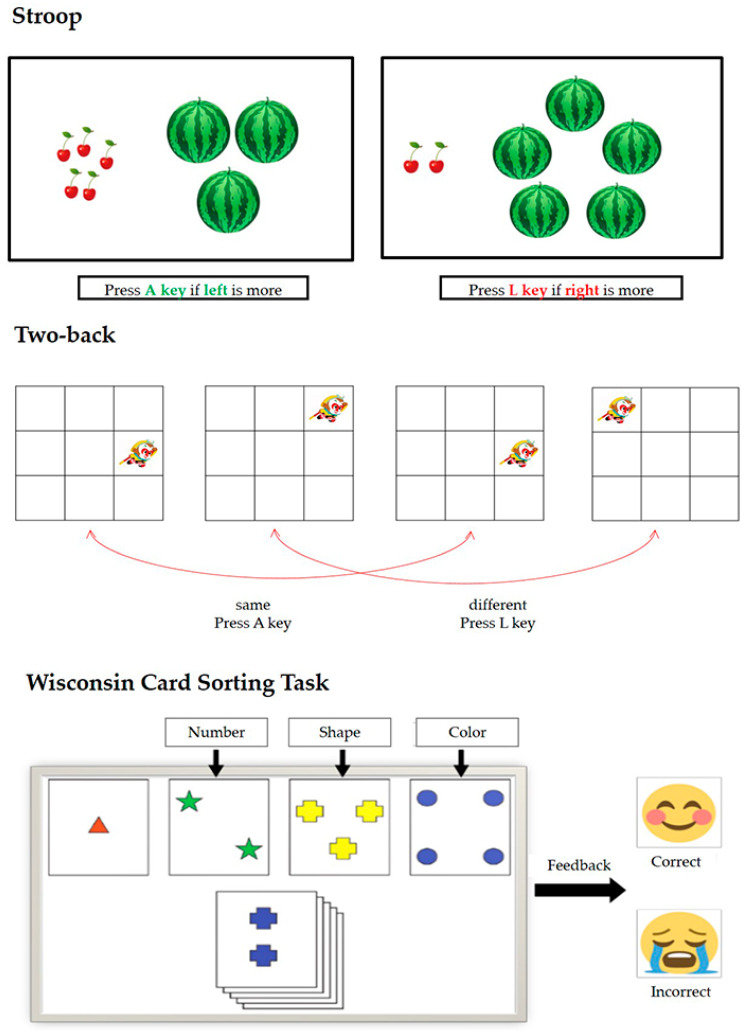
Experimental procedure for assessing executive function.

Figure 1 shows a detailed description of the experimental protocol for assessing executive function.

#### 2.2.3. Depression

Depression has been clinically diagnosed by psychiatrists based on the Chapter “Mental and behavioral disorders” of the *International Statistical Classification of Diseases and Related Health Problems, Tenth Revision (ICD-10)* (World Health Organization, 2015). Participants in the healthy group completed the Self-rating Depression Scale and the scores were all lower than 53, which suggested no depression (Zung, 1965). Therein, adolescents with depression are coded as 1, while those in the healthy group are coded as 0.

### 2.3. Statistical Analysis

First, descriptive statistics and correlation analysis were performed by SPSS software (version 25). The False Discovery Rate (FDR) correction for multiple comparisons was conducted to the *p*-values of the correlation analyses in order to control the proportion of false discoveries in multiple comparisons. Second, Mplus software (version 8.3) was utilized for mediation analysis. The model included three parallel mediators: working memory, inhibition, and shifting, which allowed us to examine specific indirect effects through each mediator as well as the total indirect effect. The missing values of measurement items or tasks were relatively low (0.02%). The missing data were completely random, as confirmed by Little’s Missing Completely At Random (MCAR) Test. To handle the missing data, the full information maximum likelihood (FIML) method was employed, which ensured robust parameter estimation. As depression is a binary outcome, the weighted least squares mean and variance adjusted (WLSMV) estimator was used, as it is specifically designed for categorical outcomes (Nguyen et al., 2016). The significance of indirect effects was assessed using the Bootstrap method with 5000 resamples, which generated an empirical distribution of indirect effect estimates. To account for multiple comparisons across the mediation pathways, confidence intervals (CIs) were adjusted using the FDR correction method. A corrected 95% confidence interval that did not include zero was considered statistically significant. This approach ensured rigorous and reliable evaluation of the mediating effects. Good model fit was indicated if the comparative fit index (CFI) was greater than 0.90 and if the root mean square error of approximation (RMSEA) was less than 1.00 (Hu & Bentler, 1999). Finally, the sensitivity analysis was employed to evaluate the robustness of the cross-sectional mediation model (Georgeson et al., 2023).

## 3. Results

### 3.1. Descriptive Statistics and Correlation Analysis

The descriptive statistics and correlations between the variables are presented in Table 1. Stressful life events showed a positive correlation with adolescents’ depression, while stressful life events were negatively linked with both working memory and inhibition. Depression is negatively associated with working memory and inhibition. No significant correlation was found between shifting and either stressful life events or depression. Moreover, there were significant intercorrelations among most executive function tasks. The grade was positively correlated with working memory. Moreover, females reported more stressful life events. They were also more likely to be depressed. Males performed better on inhibition. Thus, grade and gender were used as covariates in the subsequent analyses.

### 3.2. Mediation Analysis

**Figure 2:**
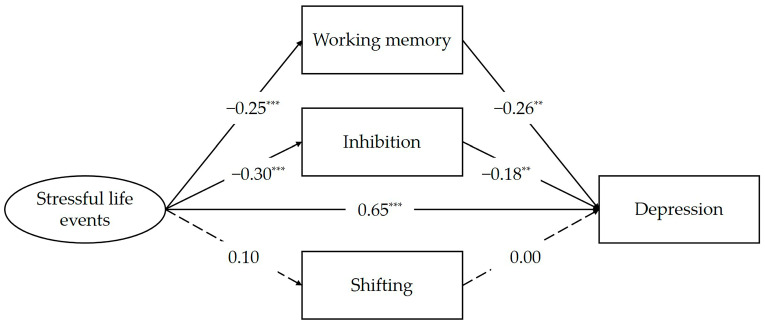
Executive functions mediated the correlation between stressful life events and depression. ** *p* < 0.01, *** *p* < 0.001.

All coefficients reported are in standardized units. The mediation model displayed an acceptable fit to the data (CFI = 0.93, RMSEA = 0.13). The result shown in Figure 2 indicates that stressful life events were positively correlated to depression (*β* = 0.65, *p* < 0.001). In addition, stressful life events were negatively related to the adolescent’s working memory (*β* = −0.25, *p* < 0.001) and inhibition (*β* = −0.30, *p* < 0.001). Working memory (*β* = −0.26, *p* < 0.01) and inhibition (*β* = −0.18, *p* < 0.01) significantly were associated with depression. Moreover, both working memory (*β* = 0.07, 95% corrected CI [0.01, 0.13]) and inhibition (*β* = 0.05, 95% corrected CI [0.00, 0.10]) partially mediated the relation between stressful life events and depression. However, shifting was not associated with either stressful life events or depression. Thus, the mediating effect of shifting was not found. The significance of the indirect effects is shown in Table 2.

### 3.3. Sensitivity Analysis

**Figure 3:**
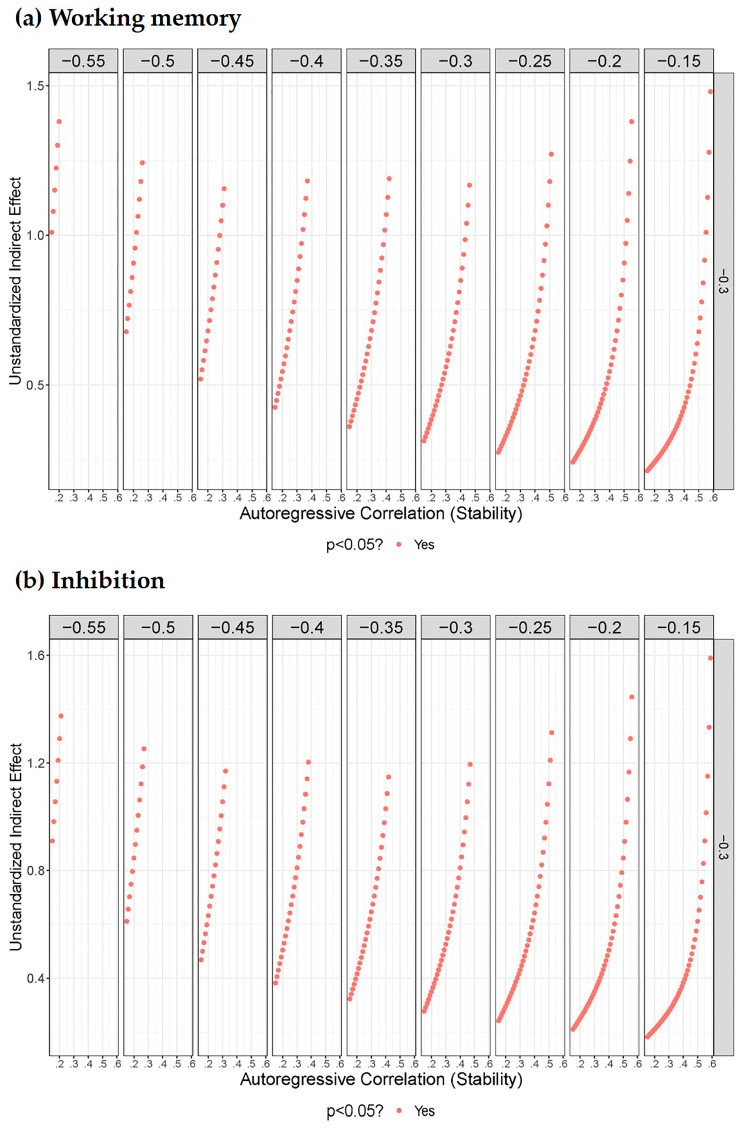
Sensitivity analysis plots for the mediating effects of (**a**) working memory and (**b**) inhibition, controlling for autoregressive and cross-lagged correlations. The maroon points indicate statistically significant estimates of the indirect effect.

Cross-sectional mediation is unable to control confounders including autoregressive and cross-lagged correlations, which might result in a bias for the mediating effect. Thus, we conducted a sensitivity analysis to assess how the mediated effect could change when different autoregressive and cross-lagged values are adjusted. Time1 variables are the phantom variables, and the Time2 variables are the “original” variables (Georgeson et al., 2023). The values from 0.15 to 0.70 (from weak to strong correlation) were used for the autoregressive correlations (*r* _(Time1 working memory, Time2 working memory)_, *r*_(Time1 inhibition, Time2 inhibition)_, and *r*_(Time1 depression, Time2 depression)_). The values from −0.15 to −0.70 were employed for the cross-lag correlations as well (*r* _(Time1 working memory, Time2 depression)_ and *r*_(Time1 inhibition, Time2 depression)_). The baseline correlation between Time1 working memory and Time1 depression was fixed at −0.30 (based on the *r* _(Time2 working memory, Time2 depression)_ = −0.32). The baseline correlation between Time1 inhibition and Time1 depression was also fixed at −0.30 (based on the *r* _(T2 inhibition, T2 depression)_ = −0.32). Upon accounting for the influence of confounding biases, regardless of their specific values, the mediating effects of Time2 working memory and Time2 inhibition were found to be consistently significant and positive (in Figure 3). Therefore, the mediating role in this study successfully withstands the sensitivity analysis, demonstrating its robustness.

## 4. Discussion

To understand the cognitive mechanisms underlying stress-related depression and help the diagnosis and treatment for research and practical purposes, the present study aimed to identify the different roles of diverse cognitive components of executive function in the stress–depression link. Two hundred and thirteen adolescents were recruited and tested on measures of life events, the ability to work memory, inhibition, and shifting by using a two-back task, a Stroop task, and a Wisconsin Card-Sorting task, respectively. We found that stressful life events positively correlated with adolescents’ depression, while stressful life events negatively linked with both working memory and inhibition. Depression was negatively associated with working memory and inhibition. No significant correlation was found between shifting and either stressful life events or depression. Further mediation analysis showed that both working memory and inhibition mediated the link between stressful life events and adolescent depression, while shifting did not show a mediating effect.

First, the correlation between stressful life events and adolescents’ depression echoes previous findings. Our work provides further support for the diathesis–stress model (Fox et al., 2010; Hammen, 2005, 2018; Nyarko et al., 2020; Stikkelbroek et al., 2016; Wei et al., 2022). Stressful life events increase considerably in adolescence, and adolescence is characterized by heightened vulnerability due to the brain being particularly sensitive to the negative effects of stress during this period (Fuhrmann et al., 2015). Any single stressor or multiple stressful life events, such as parental separation or divorce, family bereavement, academic failures, and residence relocation or school transfer, might exacerbate negative emotional states and feelings of distress, and in turn result in the development of depressive symptoms (e.g., Compas, 1987; March-Llanes et al., 2017; McMahon et al., 2020).

Further, our work tested whether there are characteristic deficits that are specific to either working memory, inhibition, or shifting in the stressful life events–depression link. We found that both working memory and inhibition mediated the link between stressful life events and adolescent depression. That is, once adolescents are faced with stressful life events, they will demonstrate lower levels of working memory and inhibitory control, which in turn will cause depression. It might be that under stress, adolescents are prone to keep negative contents in the working-memory system, have difficulties controlling these contents’ influence on their emotion regulation or manipulating their negative effect, and/or have negative biases in attention or memory (e.g., Joormann & Tanovic, 2015). These cognitive processes lead to the onset and development of depressive episodes (LeMoult & Gotlib, 2019).

However, there is no association between stressful life events and shifting or depression and shifting; the ability to shift did not play a mediating role in the relationship between stressful life events and depression. A possible reason might be that the development of shifting starts later than working memory and inhibition and continues throughout adolescence and into adulthood, peaking at the age of 21–30 (Gabrys et al., 2017; Huizinga & Van Der Molen, 2007). Its maturational development might buffer the decline in abilities caused by stressful life events among adolescents. Thus, if shifting skills are not fully developed, there might be a floor effect in the Wisconsin Card-Sorting Task, which led to a restricted range of variation in shifting abilities and thus failed to provide enough predictive power for depression (Fukuzaki & Takeda, 2022). This was supported by a meta-analysis showing that the relationship between shifting and depression is stronger in adults (Snyder, 2013). It might be due to that fully matured shifting abilities provide better support for emotional regulation in later stages of development. Another possibility is that working memory directly supports the retention and manipulation of positive information critical for regulating emotions, solving problems, and avoiding negative thought patterns, while inhibitory control plays a direct role in suppressing inappropriate or negative reactions, helping individuals interrupt ruminative cycles (Snyder, 2013). However, shifting primarily involves transitioning between thoughts or avoiding negative emotions rather than directly addressing emotional processes, which may weaken its predictive role in depression.

In all, our findings highlighted the significant roles that working memory and inhibition play in the relationship between stressful life events and depression. Our results underscore the importance of integrating cognitive training programs that specifically enhance working memory and inhibitory control into preventive mental health interventions (Beloe & Derakshan, 2020). By improving inhibitory control, patients may be better equipped to manage intrusive negative thoughts or ruminative tendencies, which are common in depression. Strengthening working memory could further support individuals in maintaining attention to positive coping strategies.

Although the current study has many strengths, it should be viewed in light of several limitations. First, our participants were recruited from a single restricted region, which may limit the generalizability of the findings. Regional differences in cultural, educational, and socioeconomic factors could influence adolescents’ cognitive function and stress responses (Xu et al., 2020; Erath & Pettit, 2021). Future research would benefit from incorporating samples from diverse regions or nationally representative populations to improve external validity. Second, part of our data was collected during the COVID-19 pandemic. The pandemic significantly disrupted daily routines and behaviors, leading to social isolation, heightened perceived stress, and decreased physical activity. These changes may influence cognitive function, increase the prevalence of anxiety and depression, and affect participant recruitment, thereby introducing additional variability into our findings. Evidence has shown that COVID-19 infection is linked with cognitive deficits and depressive symptoms as well (Hampshire et al., 2024; D’Hondt et al., 2024). Third, although we included gender as a covariate in the analysis to control for its potential influence on the results, we call for a more balanced and diverse sample in terms of gender and other relevant demographics in future studies to further investigate and validate the present findings. Fourth, this study did not account for the influence of temperament traits, which play a critical role in shaping cognitive and emotional responses to stress (Favaretto et al., 2024). These traits may significantly interact with executive function and stressful life events, influencing the development and progression of depression in adolescents. Incorporating temperament into future studies would offer a more nuanced and comprehensive understanding of how individual differences contribute to these relationships, ultimately improving the effectiveness of targeted interventions. Fifth, our study concentrated on a specific age group, which may limit the generalizability of the findings to other developmental stages. Future research should include diverse age groups to explore whether similar relationships between stressful life events, executive function, and depression are present across different stages of development. Finally, a cross-sectional design might limit us in drawing a causal conclusion. To validate and strengthen our findings, future research should incorporate longitudinal designs to examine the stability and directionality of the observed associations.

## 5. Conclusions

In summary, our study clarified the mediating roles of executive function subcomponents in the relationship between stressful life events and depression in Chinese adolescents. Specifically, working memory and inhibition mediated this relationship while shifting did not. These findings shed light on the psychological mechanisms underlying adolescent depression and emphasize the importance of targeting specific executive functions in prevention and treatment. Cognitive training programs that enhance working memory and inhibitory control may help mitigate the impact of stress on adolescent mental health. Future studies should combine both cross-sectional and longitudinal designs to assure the generalizability of our findings and the causal pathways, eventually providing rationale for optimal intervention strategies and promoting the mental health of adolescents.

## Figures and Tables

**Table 1:** Descriptive statistics and correlation coefficients of variables.

Variable	1	2	3	4	5	6	7
1 Gender	–						
2 Grade	−0.06	–					
3 Stressful life events	−0.25 ***	0.01	–				
4 Working memory	0.09	0.31 ***	−0.25 **	–			
5 Inhibition	0.18 *	0.03	−0.30 ***	0.21 *	–		
6 Shifting	0.02	0.14	0.12	0.27 ***	0.06	–	
7 Depression	−0.34 ***	−0.00	0.72 ***	−0.32 ***	−0.32 ***	0.05	–
*M*	0.47	9.34	2.58	0.82	696.75	5.93	0.54
*SD*	0.50	1.33	0.98	0.12	131.72	4.30	0.50

*Note.* * *p* < 0.05, ** *p* < 0.01, *** *p* < 0.001. *p*-values were adjusted using the FDR correction method. Gender is dummy coded with male = 1 and female = 0, and the mean represents the proportion of males. Grades refer to both junior school and high school (grades 7–12).

**Table 2:** Bootstrap analysis for the significance test of mediating effect.

Model Pathway	Estimate	SE	95% CI	
			Lower	Upper
Direct effect	0.65	0.09	0.50	0.81
Total indirect effects	0.12	0.05	0.01	0.23
Stressful life events → Working memory → Depression	0.07	0.03	0.01	0.13
Stressful life events → Inhibition → Depression	0.05	0.03	0.00	0.10
Stressful life events → Shifting → Depression	0.00	0.01	−0.00	0.00

*Note.* CI = Confidence Interval; 95% CI was adjusted using the FDR correction method.

## Data Availability

The data presented in this study are available on request from the corresponding author due to concerns regarding patient privacy and confidentiality.
